# Stem Cell-Based Therapies in Autoimmune Diseases: Current Evidence, Unmet Needs, and Future Directions—A Closing Editorial Review

**DOI:** 10.3390/cells15040328

**Published:** 2026-02-11

**Authors:** Györgyi Műzes, Ferenc Sipos

**Affiliations:** Immunology Division, Department of Internal Medicine and Hematology, Semmelweis University, Szentkirályi Street 46., H-1088 Budapest, Hungary

**Keywords:** autoimmune diseases, stem cell therapy, secretome, immune reset, immune modulation

## Abstract

The long-lasting, varied, and complicated nature of immune system issues in autoimmune disorders continues to make treatment difficult. Although standard immunosuppressive and biologic therapies have enhanced disease management, they infrequently provide enduring remission and often result in cumulative damage. Due to this, stem cell treatment has emerged as a potential alternative that aims to restore immunological homeostasis rather than maintain long-term immune suppression. This editorial review provides a comprehensive overview of the current evidence, unmet requirements, and future directions in the field, summarizing the primary contributions of the Special Issue “Stem Cell Therapy for Autoimmune Diseases”. We examine the conceptual distinction between immune reset, as demonstrated by hematopoietic stem cell transplantation, and immune modulation, which is facilitated by mesenchymal stromal cells and their secretome. Systemic sclerosis, neuroimmunological disorders, inflammatory bowel disease, and type 1 diabetes exhibit disease-specific clinical experiences that underscore both context-dependent limitations and therapeutic potential. Meanwhile, an urgent need to address persistent issues such as incomplete immune reconstitution, autoreactive memory cell-driven relapse, a lack of predictive biomarkers, safety concerns, and complex ethical and regulatory problems is addressed. This review concludes by offering perspectives on the future development of this approach, highlighting standardization, biomarker-driven patient selection, and next-generation techniques, including extracellular vesicles and genetically modified cells. This overview marks stem cell therapy as a crucial area of research for the treatment of autoimmune disorders.

## 1. Introduction

Autoimmune diseases (ADs) represent a heterogeneous group of chronic inflammatory conditions characterized by a breakdown of self-tolerance and sustained immune dysregulation. Though they continue to pose a major therapeutic challenge due to their chronicity, heterogeneity, and complex immunopathogenesis, conventional and biologic immunosuppressive therapies have markedly enhanced disease management [1,2,3,4,5]. However, these approaches frequently do not achieve sustained remission, especially in patients with aggressive or treatment-resistant disease trajectories [6,7,8,9,10,11]. Moreover, long-term immune suppression is frequently accompanied by substantial toxicity [12], infection risk [13,14], fractures [15], and malignancy [16,17], which points to the need for alternative strategies that restore immune balance rather than perpetuate immune inhibition.

The Special Issue of *Cells* titled “Stem Cell Therapy for Autoimmune Diseases” seeks to provide a comprehensive and well-balanced analysis of the current state of stem cell-based therapies in the treatment of autoimmune diseases. Comprising explorations of mechanistic insights, translational challenges, and clinical experiences from a wide range of disease entities, the contributions, as a whole, highlight the positive aspects of these methods as well as the considerable shortcomings that must be addressed before they can be practically applied in a wide range of situations.

## 2. Immune Reset Versus Immune Modulation: Mechanistic Foundations with Clinical Implications

One of the central conceptual themes emerging from this Special Issue is the distinction between immune “resetting” and immune “modulation”. Hematopoietic stem cell transplantation (HSCT), extensively discussed in the context of systemic sclerosis [18,19], Crohn’s disease [20], and other severe autoimmune conditions [21,22], exemplifies the former. This approach offers the possibility of long-term illness remission through the re-establishment of central and peripheral tolerance mechanisms, and is achieved by ablating and reconstituting the immune system.

Complementing these disease-specific analyses, broader overviews of cellular therapies in systemic sclerosis illustrate the ongoing evolution from conventional HSCT toward innovative and potentially safer cellular strategies. These include modified conditioning protocols [23,24,25], alternative cell sources [26,27,28], and combination approaches, reflecting a field in active transition rather than clinical maturity.

However, the risks associated with HSCT continue to strongly impact its therapeutic success. The papers in this Special Issue highlight the fact that although mechanistic studies have shown a dramatic reconfiguration of the T and B cell repertoire, regulatory cell compartments, and cytokine networks, these immunological benefits need to be evaluated against the morbidity and mortality associated with treatment. As a result, HSCT is now considered a therapeutic option for a carefully selected patient group, rather than a treatment that can be widely applied.

The mechanistic paradigm represented by mesenchymal stromal/stem cells (MSCs) and their derivatives (i.e., secretome), on the other hand, is fundamentally different. MSCs act as dynamic immunoregulatory agents rather than a replacement for the immune system. Their effects are exerted via soluble mediators, extracellular vesicles, and metabolic reprogramming, thereby influencing both the innate and adaptive arms of the immune system. Some publications discussing the biology of MSCs [29], secretome [30], and donor-related variability [31] highlight the fact that these cells and cellular derivatives function less as static therapeutic products and more as sensitive biological systems.

From a clinical standpoint, this trait raises significant issues related to standardization, dosage, and the predictability of response. The variability stemming from donor age, sex, tissue source, and manufacturing conditions presents a significant obstacle to the progress of MSC therapies designed for universal application. Instead, this diversity suggests that a tailored or disease-specific strategy might be more appropriate.

## 3. Disease-Specific Contexts: Lessons from Clinical Experience

This Special Issue highlights the fact that the therapeutic value of SC-based interventions is highly dependent on the context in which they are administered by covering a wide range of autoimmune and immune-mediated inflammatory diseases. These diseases include systemic sclerosis, neuroimmunological disorders, inflammatory bowel disease, and type 1 diabetes mellitus.

Neuroimmunological illnesses are a prime example of the combined problem of immune dysregulation and limited regenerative capacity [21]. In these cases, stem cell therapies need to be studied not only for their immunomodulatory efficacy but also for the possible neurological dangers they may pose [32,33].

In a similar vein, the question that needs to be answered in the case of type 1 diabetes is not simply whether immunological tolerance can be restored; rather, it is whether intervention takes place at an early enough stage to either preserve or productively regenerate functional tissue [22,34,35,36].

The absence of validated biomarkers capable of consistently guiding patient selection, predicting treatment response, and monitoring long-term immune reconstitution is a recurrent theme seen across these various therapeutic contexts. Throughout the entirety of the Special Issue, this is one of the most substantial translational gaps that was discovered.

## 4. Persisting Knowledge Gaps and Challenges

Even while the results are promising, the information provided in this Special Issue shows that the use of stem cell therapy for ADs is still very questionable. Some important questions remain to be answered, including how immune reconstitution and tolerance induction work, how long clinical responses last, and how accurate biomarkers that can predict efficacy and toxicity can be found.

Despite some patients showing clinical improvement, the process of immune reconstitution after autologous HSCT is still not fully realized and presents several problems that remain to be solved [37,38,39]. The recurrence of disease in certain individuals indicates that pathogenic autoreactive memory cells may not be eradicated by conditioning regimens or that enduring genetic and environmental variables perpetuate autoimmunity. Immune reconstitution, especially in the T-cell compartment, is frequently gradual and uneven, with complete recovery necessitating one to two years. The early recovery of T-cells after a transplant is largely dependent on the homeostatic proliferation of mature lymphocytes that survive conditioning, not on new thymic production. This procedure could therefore allow autoreactive clones to remain or even grow. Additionally, the lack of dependable biomarkers capable of forecasting long-lasting immunological tolerance makes it difficult to evaluate immune “resetting” in real time [37,38,39]. New research suggests that memory stem T cells may play a role in illness recurrence, as these cells can survive depletion and then go on to create new immune populations that do not work properly [40].

In terms of mechanics, immune reconstitution following HSCT entails a dramatic change in the immune system [41,42,43]. High-dose conditioning regimens, usually consisting of cyclophosphamide and anti-thymocyte globulin, cause severe lymphodepletion, which does away with most of the autoreactive immune repertoire that was already there. Innate immune cells, such as neutrophils, monocytes, and natural killer cells, recover swiftly—typically within the initial month—while adaptive immune reconstitution is significantly postponed [41,42,43]. Thymic regeneration is what makes long-term immunological recovery possible. It slowly recovers thymopoiesis and creates a new and varied T-cell receptor repertoire [44]. This late stage of reconstitution is crucial for making the immune system mostly naïve and generally tolerant of self-antigens [41,42,43,44].

The primary aim of stem cell-based therapy, in addition to immunological reconstitution, is the active promotion of immune tolerance rather than prolonged immunosuppression [45,46]. HSCT has consistently been linked to the reconstitution of regulatory T-cell populations, notably FoxP3-expressing Tregs, which are crucial in inhibiting autoreactive immune responses. Additionally, qualitative reprogramming of residual autoreactive T cells has been documented; for example, studies in juvenile idiopathic arthritis have demonstrated a phenotypic shift from a pro-inflammatory, IFN-γ-dominant profile to a more tolerogenic state characterized by IL-10 and GATA-3 [47]. At the same time, the B-cell compartment undergoes many changes, leading to the creation of a new repertoire lacking any autoreactive clones that cause disease [48]. All of these processes work together to restore immunological homeostasis and support the long-term therapeutic promise of HSCT in autoimmune disorders, despite challenges remaining in achieving long-lasting and universal tolerance.

An additional problem is that most of the available data come from small, heterogeneous cohorts, retrospective analyses, or early-phase clinical trials. Direct comparisons between different cellular products, conditioning regimens, or disease stages are largely lacking. Regulatory [49,50], ethical [50], and economic considerations further complicate the translation of SC-based therapies into routine clinical practice.

As a “living drug”, cell-based therapy is complicated and heterogeneous. Tumorigenicity is essential for a safety assessment of cell-based therapy [49,50]. The end output of stem cell-based therapies may contain leftover undifferentiated cells with significant proliferation and differentiation potential, which could cause tumors in vivo. Source, phenotype, differentiation status, proliferative capacity, ex vivo culture conditions, ex vivo processing procedures, injection site, and administration route also affect cell tumorigenicity. The complexity of its design, as well as many other elements, must be considered while assessing tumorigenicity. A partial review and overview of products on the market and in development, as well as actual experience of use, shows that cell tumorigenicity requirements and practices vary globally [49]. The need for guidance and support for applicants’ declaration requirements in different locations should be addressed alongside product attributes and regulatory requirements. No global regulatory consensus exists on technical implementation guidelines, and evaluation methods lack quantitative or standardized specifications.

The ethical implications of stem cell research are often discussed in terms of therapeutic usefulness, safety, adverse effects, and risks—so-called “hard impacts” [51,52] —which are usually quantified. However, to understand the ethical consequences of stem cell research for science and society, soft repercussions must also be considered. Stem cell research often has indirect implications on behavior, experiences, actions, morality, and societal systems. Hard and soft impacts provide a more complete view of stem cell research’s social and ethical ramifications and can help make the profession more convivial. Soft impacts help researchers understand the wide range of significant ramifications of their work, and acknowledging these repercussions is equally essential for the honest exploration of the ethical and societal ramifications of stem cell research [51,52].

In the past few years, the market for stem cell therapy has expanded at an exponential rate [53]. The global stem cell market size was valued at US$297 million in 2022 and is expected to display a compound annual growth rate of 16.8% from 2022 to 2027, as evidenced by the results of the stem cell market growth rate. This growth is driven by factors such as increasing funding for stem cell research, the rising demand for regenerative medicine, the growing number of technologies and facilities for cell therapy, and ongoing clinical trials with promising results [53]. To further develop the market, policymakers and regulatory bodies must streamline the intricate process of obtaining regulatory approvals for clinical use [53,54]. Nevertheless, there is growing concern regarding the escalating number of unapproved stem cell treatments.

## 5. Future Perspectives

As we look to the future, the convergence of basic immunology, systems biology, and clinical research will be essential for the advancement of this discipline. The goals of future research should be to (i) improve patient stratification in order to determine which patients are most likely to benefit from SC-based therapies; (ii) standardize manufacturing, dosing, and monitoring methods; and (iii) establish reliable biomarkers to guide treatment decisions and long-term follow-up.

Equally essential is the exploration of next-generation techniques, including genetically edited stem cells [55,56,57], combination therapy with biologics or small drugs [58], and cell-free products such as extracellular vesicles [59,60,61,62]. It is possible that these solutions will offer enhanced safety, scalability, and precision, bringing stem cell therapy closer in line with the concepts of personalized medicine.

## 6. Concluding Remarks

In summary, this Special Issue provides a multidimensional and integrative overview of the use of stem cell-based therapies for the treatment of autoimmune diseases, highlighting both their transformative potential and their current scientific, translational, and clinical limitations. By bringing together mechanistic insights, preclinical evidence, and clinical perspectives across a wide range of autoimmune disease settings, the collected contributions collectively advance the field and offer a structured roadmap for future research and therapeutic development.

This Special Issue was designed to create a conceptual framework for the future development of stem cell-based therapy techniques, in addition to documenting the existing state of the art. The contributions consistently emphasize that, although stem cell therapies are unlikely to supplant traditional immunosuppressive or biologic treatments in the immediate future, they possess a distinctive and persuasive potential to fundamentally alter disease trajectories by addressing the underlying mechanisms of immune dysregulation and reinstating immune homeostasis.

Even though this type of therapy presents a promising area for further research, many problems remain to be solved, such as finding the best sources of cells, improving manufacturing and standardization procedures, choosing the right patients, ensuring long-term safety, and finding accurate indicators of response. To solve these problems, key scientists, translational researchers, and clinicians will need to work together closely in the long term, whilst also utilizing new technology and systems-level approaches.

We hope that this Special Issue will be useful not only as a resource for understanding the present state of stem cell-based treatments for autoimmune diseases but also as a means of encouraging the development of new ideas and conversations between different fields. Cellular therapies focused on immune reprogramming may persist as one of the most inventive and intellectually stimulating areas in the management of autoimmune diseases by promoting logical, evidence-based, and accountable progress.

## Data Availability

No new data were created.
